# Essure Surgical Removal and Subsequent Resolution of Chronic Pelvic Pain: A Case Report and Review of the Literature

**DOI:** 10.1155/2016/6961202

**Published:** 2016-01-24

**Authors:** Isamarie Lora Alcantara, Shadi Rezai, Catherine Kirby, Annika Chadee, Cassandra E. Henderson, Malvina Elmadjian

**Affiliations:** ^1^Department of Obstetrics and Gynecology, Lincoln Medical and Mental Health Center, Bronx, NY 10451, USA; ^2^West Virginia School of Osteopathic Medicine (WVSOM), Lewisburg, WV 24901, USA

## Abstract

*Background*. Hysteroscopic tubal sterilization (Essure) is a minimally invasive option for permanent contraception with high reported rates of patient satisfaction. A small percentage of these women subsequently choose to have the tubal inserts removed due to regret or perceived side effects such as late-onset pelvic pain secondary to placement of the Essure device.* Case*. A twenty-nine-year-old woman G4P4014 presented with a two-year complaint of chronic pelvic pain and dyspareunia after the hysteroscopic placement of an Essure device for sterilization. On reviewing the images of the HSG, it was noted that although tubal occlusion was confirmed, the left Essure coil appeared curved on itself in an elliptical fashion and did not seem to follow the expected anatomic trajectory of the fallopian tube. The patient reported resolution of chronic pelvic pain following laparoscopic removal of Essure device.* Conclusion*. A misplaced Essure device should be considered in the differential diagnosis of chronic pelvic pain in women who had difficult placement of the device. In addition to demonstrating tubal occlusion, careful examination of the configuration of the Essure microinserts on HSG examination provides valuable information in patients with pelvic pain after Essure placement.

## 1. Background

Essure hysteroscopic tubal sterilization system (Conceptus Inc., Mountain View, CA) [1, 2] is a sterilization device that consists of an expanding microinsert that is placed into the cornual section of the fallopian tube during hysteroscopy [3]. The initial five-year placement success rates by tubal occlusion ranged from 84% to 99.8% [3]. Essure requires a confirmation of proper placement with a follow-up hysterosalpingogram (HSG) at three months [4, 5].

Essure is a minimally invasive option for permanent contraception with high reported rates of patient satisfaction [3, 6–9]. A small percentage of women subsequently choose to have the tubal inserts removed due to regret or perceived side effects [3]. Complications associated with the Essure device include improper placement (malpositioning), unintended pregnancy [10], chronic pelvic pain, infection, and nickel allergy [6]. There is limited information with regard to the improvement in the symptom profile following surgical removal of the tubal inserts [3].

## 2. Presentation of the Case

The patient is a 29-year-old G4P4014 who delivered and elected on discharge to use oral contraceptive pills (OCPs) until scheduled for an interval postpartum tubal sterilization. The initial attempt to place the Essure device failed, because the fallopian tube ostia were not visualized. One month later, placement of the Essure procedure was successful. There were three coils seen at the ostia of the right fallopian tube and one coil seen in ostia of the left tube. The patient tolerated the procedure well and went home the day of the procedure. She had no symptoms and no complaints at the two-week postprocedural follow-up visit and the evaluation was unremarkable. Patient did not have any history of gonorrhea or chlamydia infection, nor any history of pelvic pain or pelvic inflammatory disease (PID) prior to Essure placement.

**Figure 1:**
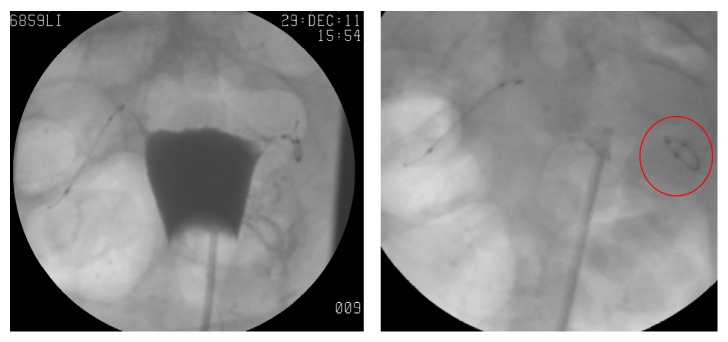
Post-Essure hysterosalpingogram (HSG) (12/29/11): no spillage is noted on either side, which confirms bilateral tubal occlusion, while showing the left Essure coil microinsert twisted in an abnormal configuration (red circle), which does not seem to follow the expected anatomic trajectory of the fallopian tube. Instead, it appears curved on itself in an elliptical fashion.

Patient did not keep her appointment for the three-month interval hysterosalpingogram (HSG) follow-up for evaluation of tubal patency; instead, the HSG was done six months after Essure placement and confirmed successful tubal occlusion (Figure 1).

**Figure 2:**
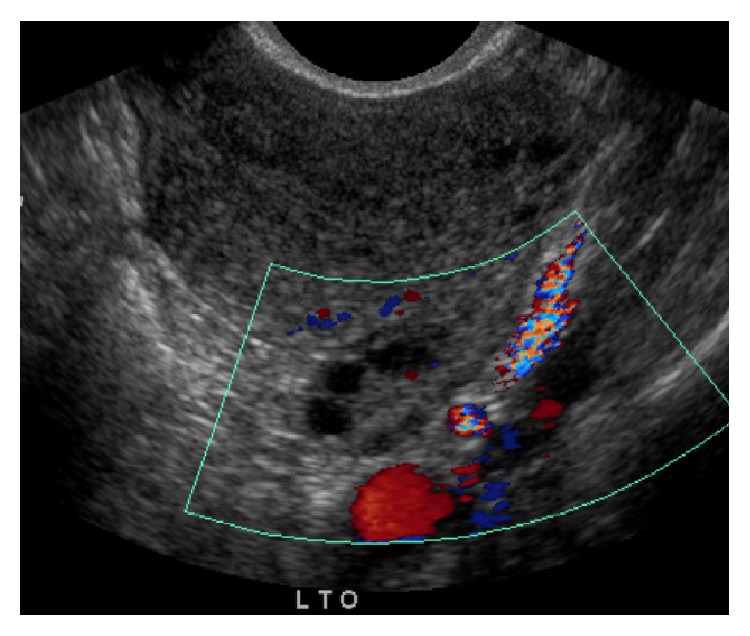
Pelvic ultrasound 5/31/13: unremarkable scan, picture showing normal left ovary with the normal Doppler flow.

Approximately two years after the successful placement of the Essure device, the patient presented with lower abdominal pain that she reported began gradually and became more frequent after the Essure was placed. The patient described the pain as constant and exacerbated during intercourse (dyspareunia). It was not related to menses initially, but overtime it began to get worse during menstruation as well. During in-office gynecologic examination, there was no cervical motion tenderness or significant right adnexal findings. However, there was left adnexal tenderness elicited during the examination. A normal uterus and bilateral adnexa with no free fluid were identified on pelvic imaging (Figure 2). Gonorrhea and chlamydia screening remained negative. A diagnosis of left parametritis was made for which a 14-day course of doxycycline was given. A three-week follow-up evaluation with pelvic ultrasound was scheduled.

At the three-week follow-up, the patient reported no resolution of symptoms and requested removal of the Essure device. Patient underwent laparoscopic bilateral total salpingectomy with bilateral Essure inserts removal. Laparoscopically, left fallopian tube was grasped with the forceps; the Essure device was identified at the isthmus and ampullary portion of the fallopian tube and small incision was made to expose the Essure device distal tip. The inner and outer coils of the Essure were removed in two complete pieces using the grasping forceps and then fallopian tube was excised completely using LigaSure device. The same procedure was performed on the contralateral side and Essure device and fallopian tubes were removed bilaterally.

Intraoperative finding was notable for normal fallopian tubes and ovaries, without any evidence of endometriosis, adhesions, or any other pelvic pathology. The patient reported resolution of chronic pelvic pain following surgical removal of Essure device. On reviewing the images of the HSG, it was noted that although tubal occlusion was confirmed, the left Essure coil appeared twisted in an abnormal configuration and did not seem to follow the expected anatomic trajectory of the fallopian tube. Instead, it appeared curved on itself in an elliptical fashion (Figure 1).

At the one-month postoperative and subsequent interval gynecology visits, the patient reported complete resolution of presenting pelvic symptoms.

## 3. Discussion

The Essure device is a noninvasive, permanent option for sterilization. It is inserted in the office, does not require general anesthesia, and has been shown to be more cost effective than laparoscopic sterilization [11]. It also does not require an incision and has a lower incidence of postoperative pain compared to surgical sterilization techniques [12, 13]. The average time for the procedure is under ten minutes [14]. The procedure consists of placing the small, flexible Essure microinserts into the fallopian tubes via a catheter through the vagina and cervix. The inserts are made of polyethylene terephthalate (PET) fibers that cause an inflammatory reaction resulting in fibrosis, which occludes the fallopian tubes. They also contain a flexible stainless steel inner coil and a dynamic outer nickel titanium alloy coil that expands to help anchor the device while fibrosis is occurring [14]. Three months after the device is inserted, it is recommended that the patient undergoes a hysterosalpingogram to confirm that the fallopian tubes are blocked [15]. Until this confirmation is performed, the patient must use a back-up form of birth control.

The reported complications associated with the device include heavy periods, irregular menses, dyspareunia, and spotting with ovulation [16, 17]. Other rare complications include tubal or uterine perforation, intraperitoneal migration and unintended pregnancies [10], device expulsion, and infection. One study claims that the incidence of chronic pelvic pain requiring opiates for relief following hysteroscopic sterilization versus laparoscopic sterilization is increased by eight percent [18]. Laparoscopic removal of Essure microinserts can improve patient's symptoms [3, 4, 8].

Preoperative preparation for Essure placement includes discussing all alternatives to sterilization with the patient, efficacy of the procedure, and possible complications. Like laparoscopic sterilization, antibiotic prophylaxis and thromboprophylaxis are not necessary. In order for the procedure to be successful, a clear view of the tubal ostia is required. Also, the endometrium should be thin when the procedure is performed. This can be accomplished by pretreating the patient with a progestin-containing contraceptive. After pretreatment, the procedure can be performed at any time during the cycle except for during menstruation. In our case, there was difficulty visualizing the fallopian tube ostia on entry to the uterine cavity by hysteroscopy despite appropriate preparation of the endometrium with OCPs [11].

Our patient experienced chronic pelvic pain following Essure placement and chose to have the device removed while undergoing a laparoscopic bilateral salpingectomy. She reported resolution of symptoms after the Essure device was removed. This potential side effect has been reported in the literature and can be due to misplacement of the device. It has also been noted to be more prevalent in patients who had a diagnosis of preexisting chronic pain such as chronic low back pain or fibromyalgia [1]. However, up to fifty percent of pelvic pain that develops postoperatively will resolve on its own within three months.

Placement of the Essure device is like any other surgical procedure and involves learning the proper technique of inserting the device. Hysteroscopy is used to visualize the tubal ostia but problems are sometimes encountered. There can be many factors limiting visibility such as equipment failure and uterine perforation. These issues can be minimized by good surgical technique and understanding of the hysteroscopic equipment. Currently a two percent failure rate is reported, but this is mainly due to factors such as tubal stenosis or occlusion, which make placement impossible [8]. Proper placement of the microinserts is essential in preventing the patient from developing pelvic pain.

Brito et al. presented a retrospective case series of 11 women who underwent surgical removal of Essure by hysteroscopy, salpingectomy, and/or hysterectomy [3]. The predominant symptom at presentation was pain (*n* = 10; 90.91%), as well as bleeding (*n* = 6; 54.54%) and/or dyspareunia (*n* = 5; 45.45%). After surgical removal, the majority of patients (*n* = 8; 72.72%) reported an improvement in their symptoms. However, 3 (27.27%) patients continued to have persistent symptoms after surgery [3]. In addition to cases of chronic pelvic pain due to misplaced Essure microinserts, there are cases where patients had persistent postprocedure pain in the setting of appropriately placed microinserts [4, 8].

Al-Safi et al. [2] reviewed the adverse events associated with Essure procedure as well [2]. Four hundred fifty-seven adverse events were reported in the study period. Pain was the most frequently reported event (217 events [47.5%]) followed by delivery catheter malfunction (121 events [26.4%]). Poststerilization pregnancy was reported in 61 events (13.3%), of which 29 were ectopic pregnancies. Other reported events included perforation (90 events [19.7%]), abnormal bleeding (44 events [9.6%]), and microinsert malposition (33 events [7.2%]). The evaluation and management of these events resulted in an additional surgical procedure in 270 cases (59.1%), of which 44 were hysterectomies [2].

A recent study by Mao et al. [19, 20] found that patients undergoing hysteroscopic sterilization have a similar risk of unintended pregnancy but a more than 10-fold higher risk of undergoing reoperation compared with patients undergoing laparoscopic sterilization [19–21].

Essure removal in our case resulted in resolution of the patient's pelvic pain. However, patients should be counseled that Essure removal might not always result in resolution of symptoms [22, 23]. Some studies have stated that a small percentage of patients may still experience pain after removal [24–26]. This is an issue that will require further investigation in the future.

## 4. Conclusion

This report reinforces the need to consider a misplaced Essure device in the differential diagnosis of late-onset pelvic pain in women who had difficult placement of the device. In addition to demonstrating tubal occlusion, careful examination of the configuration of the Essure microinserts on HSG examination provides valuable information in patients with pelvic pain after Essure placement or cases where tubal perforation by the device is suspected. Our case reinforces besides an unusual location of a misplaced Essure device that late-onset pelvic pain should alert the physician to such complication.

Furthermore, physicians should be cautious in performing hysteroscopic sterilization in patients with a history of chronic pain and should counsel patients that pelvic pain may develop after the procedure. Before surgical removal of Essure, it is important to thoroughly discuss the risk of continuing symptoms with patients. Physicians should help patients consider all of the potential options for sterilization, including laparoscopy, and help them decide what is best for them.

Future prospective studies should focus on chronic pain as a risk factor for development of new pain after hysteroscopic sterilization.
